# Awareness and Risk Behaviors Associated with *Tribulus terrestris* (*Tt*), Dietary Supplements, and Anabolic Steroids: Evidence from an Italian Questionnaire-Based Study

**DOI:** 10.3390/nu18020253

**Published:** 2026-01-13

**Authors:** Adele Minutillo, Omayema Taoussi, Simona Pichini, Francesco Paolo Busardò, Giulia Bambagiotti

**Affiliations:** 1 National Centre on Addiction and Doping, Istituto Superiore di Sanità, viale Regina Elena 299, 00161 Roma, Italy; adele.minutillo@iss.it; 2 Department of Biomedical Sciences and Public Health, Marche Polytechnic University, 60121 Ancona, Italy; omayema.taoussi@gmail.com (O.T.); f.p.busardo@staff.univpm.it (F.P.B.); giulia.bamba@gmail.com (G.B.)

**Keywords:** *Tribulus terrestris*, protodioscin, dietary supplements, performance enhancement, fitness culture, sport nutrition, public health

## Abstract

**Background**: *Tribulus terrestris* (*Tt*) is a popular herbal supplement marketed to enhance fitness performance, despite inconclusive evidence regarding its efficacy and safety. This study aimed to investigate the prevalence of TT use, awareness, and motivations for its use among recreational athletes in Italy, helping to address the lack of empirical data describing who actually uses *Tt*, for what purposes, and with what behavioral risks. **Methods**: A cross-sectional anonymous survey was administered between May and October 2024 across Italian gyms and fitness clubs using Microsoft Forms. A total of 696 individuals initiated the questionnaire; after removal of duplicate, incomplete and ineligible entries, 510 responses were analyzed. Two indicators of *Tt* consumption were assessed: ever use and current use, with the latter designated as the primary outcome. A multivariable logistic regression evaluated predictors of current *Tt* use, entering sex, age category (18–24, 25–34, 35–44, ≥45 years), and motivation for supplement consumption. **Results**: Current *Tt* use was reported by 7.8% of respondents, while 10.5% declared ever using a *Tt*-containing product. Motivation was the only independent predictor of *Tt* consumption (*p* = 0.012). Individuals reporting performance enhancement as their primary motivation were markedly more likely to currently use *Tt*, compared with those using supplements for other purposes (adjusted OR ≈ 18.5; *p* = 0.008). Neither sex (*p* = 0.918) nor age category (*p* = 0.519) significantly predicted *Tt* use. Admission of anabolic steroid use was infrequent but was linked to online purchasing from potentially unregulated sources. **Conclusions**: *Tt* consumption in fitness settings is driven predominantly by performance-oriented expectations rather than demographic characteristics. The observed discrepancy between consumer beliefs and scientific evidence suggests a pressing need for educational interventions and regulatory vigilance in sports nutrition. Public health policies should focus on improving label literacy, strengthening consumer protection, and countering misinformation within supplement marketing environments.

## 1. Introduction

Plant-based supplements and botanical extracts are widely used by athletes as they are often promoted for their presumed ability to increase muscle mass, enhance energy availability during exercise and aid weight loss. However, the current scientific evidence does not provide significant support for effectiveness or safety of these products in sports. From a public health perspective, a critical concern arises from the pharmacological complexity of these extracts combined with their unregulated consumption. There is a need to identify any potential adverse effects that could result from their uncontrolled use, whether taken individually or in combination with other supplements or pharmacological agents. Among the most popular products in the health and fitness market, Tribulus terrestris-based supplements are widely recommended for their supposed ability to increase testosterone production, enhance libido and fertility.

*Tribulus terrestris* (*Tt*) extract, also known as “puncture vine” or “gokhru”, is a herbal supplement obtained from the leaves and roots of the herbaceous plant *Tt*, which belongs to the Zygophyllaceae family and grows in subtropical regions worldwide. Traditionally used in Chinese and Ayurvedic medicine to treat erectile dysfunction, male impotence, rheumatism, edema, hypertension and kidney stones, it has become increasingly popular in Western countries since the 1980s, particularly among athletes and bodybuilders interested in enhancing their performance [1,2,3,4,5,6,7]. *Tt* also exhibits marked stimulating activity on the central nervous system [8,9].

Commercial *Tt* products are often sold in tablet form, containing a lyophilized extract that is enriched in protodioscin, a steroidal saponin representing approximately 45% of the total extract [10,11,12,13,14]. In animal models, this compound has been shown to enhance the endogenous production of sex hormones (e.g., testosterone, luteinizing hormone (LH) and dihydrotestosterone) and to promote nitric oxide release, resulting in vasodilation [3,9,15,16,17]. In addition to being promoted for their purported antioxidant [5,18,19,20,21], and antitumor properties [13,14,22,23,24], *Tt* supplements are widely marketed as testosterone enhancers and are also known for their claimed efficacy in managing sexual health disorders in both males and females [10,16,25,26]. These products are available to anyone without a medical prescription, both through online platforms and authorized herbal product retailers [18,27].

This wide accessibility to commercial *Tt* creates a significant pharmacological paradox. To date, no regulatory restrictions have applied to their sale and production, and the World Anti-Doping Agency (WADA) [28] has not prohibited their use in sport.

Despite its popularity, there is no scientific evidence that supports the effectiveness of *Tt* in improving athletic performance [1,29,30]. While generally considered well-tolerated, the pharmacological profile of *Tt* in humans remains largely uncharacterized. The lack of rigorous pharmacokinetic and pharmacodynamic studies makes it difficult to establish safe dosage limits or predict drug-supplement interactions [31]. Notably, clinical case reports describe significant adverse events that have occurred even following occasional consumption of *Tt*-based products [2,32,33].

Consequently, the intersection of aggressive marketing, alleged high bioactive compound content, and the absence of medical supervision presents a tangible risk to the consumer population.

Given the worldwide spread of *Tt* consumption, its high content of bioactive compounds (including flavonoids, alkaloids, lignanamides, sterols, and steroidal saponins), its frequent use in both traditional medicine and sports contexts and the absence of comprehensive human studies, there is a need for updated, reliable data on its efficacy, mechanism of action, optimal dosage and frequency of intake and potential adverse effects in humans.

This study aims to evaluate the actual consumption patterns and potential misuse of *Tt* and/or protodioscin-containing supplements by analyzing data collected via an anonymous questionnaire administered to gym attendees across Italy. The primary objectives are to achieve an in-depth understanding of consumption patterns, the underlying motivations and frequency of use, as well as to evaluate the participants’ assessment of the impact on sports performance. Specifically, based on the ergogenic claims of these products, we hypothesized that *Tt* use is expected to be significantly associated with the specific goal of performance enhancement, potentially accompanied by low user awareness regarding product composition.

## 2. Materials and Methods

A cross-sectional questionnaire-based study was carried out among individuals training in Italian fitness centers between May and October 2024. Data were collected anonymously through Microsoft Forms.

### 2.1. Participants

**Figure 1:**
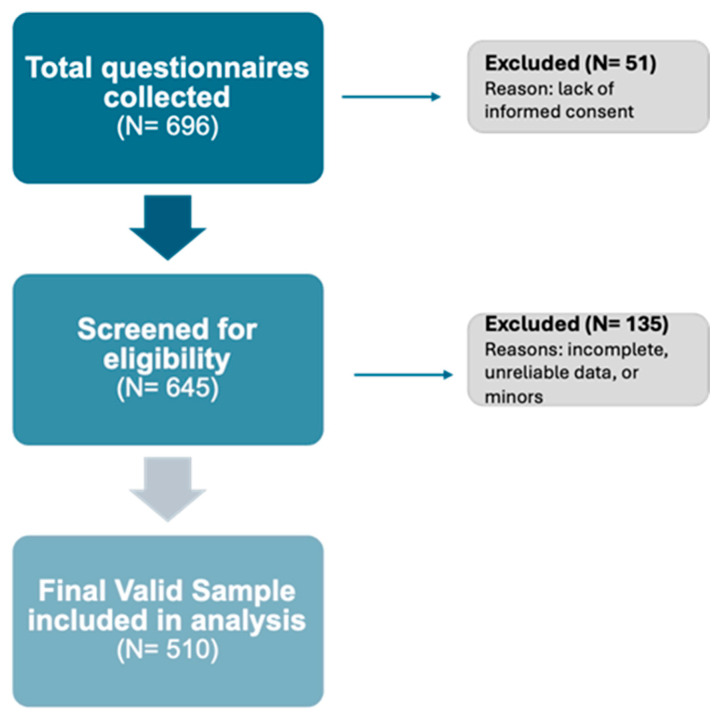
Flow chart of participants selection and data cleaning process.

Participants were eligible if they: (i) were ≥18 years old, and (ii) reported regular participation in gym-based training or structured sports activities (≥1 session per week). Exclusion criteria included incomplete survey submission, duplicate entries, and participants who did not meet eligibility criteria. recruited using a convenience sampling method between May and October 2024. A total of 696 questionnaires were initially collected. Of these, 51 were excluded: 50 due to the absence of consent and 1 due to incomplete filling, despite the participant providing consent. The remaining 645 questionnaires underwent data cleaning, following confirmation that participants had reviewed the information on personal data processing and provided informed consent. 510 (73.3% of 696 questionnaires) were retained for analysis, as illustrated in Figure 1.

### 2.2. Questionnaire

The questionnaire (available in full as Supplementary Material S1) was divided into five thematic sections containing multiple-choice questions and was designed to take a few minutes to complete. The questions covered sociodemographic characteristics (sex and age, region), anthropometric data (body weight and height). Additional sections explored details of sports practice and the use of dietary supplements in general. *Tt* intake was assessed through two separate indicators: ever use (lifetime consumption of any *Tt*-containing supplement) and current use (reported ongoing intake at the time of survey completion. Information was also requested about the use of anabolic-androgenic steroids. To reduce bias, all *Tt*-related items included brand example, generic product images, and multiple-choice descriptors. The questionnaire was adapted from validated survey forms previously employed by the Italian National Institute of Health (ISS) for national doping and supplementation surveillance campaigns.

### 2.3. Dependent and Independent Variables

The primary dependent variable was current *Tt* use (binary: yes/no). Independent variables entered into inferential analyses were sex (male, female, prefer not to answer), age group (18–24, 25–34, 35–44, ≥45 years), categorized based on recognized behavioral clusters in sports supplementation; motivation for supplement use (performance enhancement, muscle development, health/wellbeing, other).

### 2.4. Bias Control

Duplicate submissions were identified and removed by cross-checking IP addresses, device identifiers, completion times, and response patterns. The system prevented multiple entries from the same user session. The questionnaire was fully anonymous and collected no personally identifiable information. No incentives were offered.

### 2.5. Ethical Consideration

The study complied with ethical principles of the Declaration of Helsinki. Participation was voluntary, anonymous, and based on informed consent (digital and paper and pencil). Italian National Ethical Committee approved this study (prot. AOO-ISS n. 0058106 14 December 2023) and all data were collected and processed in accordance with the General Data Protection Regulation (EU Regulation 2016/679).

### 2.6. Data Collection

The questionnaire was proposed in paper and in digital format via Microsoft Forms using QR codes at 312 gyms including principal gym networks covering the entire national territory with an overrepresentation of some cities (Ancona, Rome, Florence, Palermo, and Milan). The Microsoft Forms option was implemented at the request of participating gyms and facilitated data collection and management while ensuring a higher degree of confidentiality for participants. The paper questionnaires were anonymized at source through the assignment of unique alphanumeric identification codes and subsequently shipped to the Italian National Institute of Health (ISS). Data entry was conducted independently by two trained operators. To ensure accuracy, two researchers performed systematic data entry verification, including reconciliation of discrepancies and validation of completeness and consistency of the entered records. Data cleaning procedures were then applied, covering error detection, handling of missing or implausible values, standardization of variable coding, and removal of duplicates. Finally, the cleaned dataset was harmonized with the dataset generated from the Forms digital platform through a structured database alignment process, ensuring variable format compatibility, codebook coherence, and integration into a unified analytical database.

In compliance with the General Data Protection Regulation (GDPR) and Italian privacy legislation, all questionnaires were anonymized, and each survey was assigned a randomly generated alphanumeric code. The completed questionnaires were collected and examined to determine the prevalence of dietary supplement use among athletes.

### 2.7. Statistical Analysis

Descriptive statistics (frequencies, percentages, means ± SD) characterized consumption patterns and participant attributes. Between-group comparisons were performed using Pearson’s χ^2^ tests with adjusted degrees of freedom according to the number of categories in each variable. An exploratory multivariable binary logistic regression was fitted to evaluate associated factors of current Tt use, entering sex, age category, and motivation for supplement use. Variables were entered simultaneously. Model assumptions were checked and no multicollinearity was detected. Odds ratios (OR) and 95% confidence intervals (CI) were reported. Statistical significance was set at *p* < 0.05. Analyses were performed using IBM SPSS Statistics (v.29). File inspection and exclusion were conducted prior to statistical testing. All inferential analyses were conducted using current Tt use as the outcome; lifetime and recent use were analyzed descriptively only.

## 3. Results

### 3.1. Participants

The sample of 510 individuals, belonging to 78 gyms (gym response rate: 26%), consisted of 47.1% men, 49.0% women, and 3.9% who preferred not to disclose their gender. The age distribution shows a predominance of participants in the 25–34 age group, representing more than one third of the sample (34.1%). This is followed by young adults aged 18–24 years, who account for 29.6%. The 35–44 and ≥45 age groups are less represented, with 17.3% and 19.0%, respectively. Overall, the sample is concentrated in younger and early mid-life age ranges, while older age groups contribute to a lesser extent (Table 1). Regarding anthropometric characteristics, the mean height was 172.6 cm (SD = 8.9), and the mean weight was 72.5 kg (SD = 14.0). From a geographical perspective, participants were from Central Italy (69.6%), followed by Southern Italy and the Islands (17.1%). Smaller proportions were from the North-West (6.9%) and North-East (4.9%). A very limited number of participants were from outside Italy (0.6%), and geographical area was not reported for 1.0% of the sample. Central Italy participants were primarily residents of Ancona, Rome and Florence; South and Islands participants were primarily in Palermo; and North- West participants were primarily in Milan. The remaining participants were distributed across various smaller locations throughout the country. Of all sample, 66.5% reported regularly training at the gym, while 11.7% engaged in competitive sports. Whereas 37.0% (N = 189) participants reported use of dietary supplements to improve physical appearance or performance, only 5.1% of participants (n = 26) reported using supplements containing Tt in the past six months, and 11.7% were uncertain whether their supplements contained this ingredient. Use during the previous week was even lower (n = 21; 4.1% of the total sample) (Table 2).

### 3.2. Tribulus Terrestris Users

Analyses of gender, type of sports activity, and supplement purchase channels revealed no statistically significant associations with *Tt* use, as indicated by chi-square tests (*p* > 0.05). However, a significant difference was observed regarding the reason for supplement use (χ^2^(3) = 15.13, *p* = 0.001): *Tt* was more frequently used by individuals aiming to enhance performance than by those using supplements for esthetic purposes or to compensate for nutritional deficiencies.

Independent-samples *t*-tests showed no significant differences between users and non-users of *Tt* in terms of the number of capsules or sachets consumed.

Among the total sample (N = 510), *Tt* supplement use in the past six months was reported by 5.1% (N = 26) and use in the past week by 4.1% (N = 21). *Tt* use was slightly more prevalent among males (26.2%) than among females (18.4%), with non-disclosing participants showing a similar pattern (25.0%). Males accounted for the largest share of total *Tt* users (14.0%), followed by females (7.9%) and non-disclosing participants (0.9%).

Age-stratified analysis indicated the highest prevalence in the 25–34 age group (31.7%), followed by 35–44 years (22.7%), while 18–24 and ≥45 years showed lower prevalence (14.3% and 16.7%). In terms of contribution to total *Tt* users, the 25–34 group accounted for 11.4%, and the 35–44 and ≥45 groups for 4.4% each, suggesting that *Tt* use is concentrated among participants aged 25–34 years.

Regarding supplement motivations, *Tt* use was most frequent among participants using supplements to improve performance (35.9%) or enhance physical appearance (40.0%), while those supplementing for nutritional deficiencies (2.9%) or other reasons (7.1%) reported substantially lower use. The largest contributions to total *Tt* users came from performance enhancement (12.5%) and physical appearance (8.9%).

As above reported, 11.7% participants were unsure whether their supplements contained *Tt* or protodioscin, and 7.4% did not recall the product type. Lack of awareness clustered among individuals purchasing supplements online (χ^2^(3) = 11.46, *p* = 0.009), indicating potential exposure to unlabeled or poorly labeled pharmacologically active products. *Tt* use was not significantly associated with gender, sport participation, purchase channel, or recent anabolic steroid use (all *p* > 0.12).

### 3.3. Factors Associated with Current Tt Use

A multivariable binary logistic regression was fitted to identify factors associated with current *Tt* use. Motivation for supplement use emerged as the sole independent variable. Compared with participants who reported performance enhancement as their primary motivation, those motivated by non-performance reasons—including health/wellbeing and general fitness—were significantly less likely to consume *Tt* (adjusted OR = 0.054; 95% CI: 0.006–0.461; *p* = 0.008). Neither sex (*p* = 0.918) nor age category (*p* = 0.519) predicted *Tt* use (Table 3). For interpretive clarity, the inverse OR indicates that performance-oriented individuals were approximately 18.5 times more likely to currently use *Tt* than other supplement users with different motivations.

## 4. Discussion

This investigation provides novel empirical evidence on the real-world consumption of *Tt* among Italian gym users. Our findings demonstrate that performance-oriented motivation, rather than demographic characteristics, is the predominant associated variable of current *Tt* use, with performance-focused individuals being approximately 18.5 times more likely to consume *Tt* than those using supplements for other reasons. Despite its modest prevalence, *Tt* emerges as a behavioral marker of performance-seeking attitudes, commercial susceptibility, and exposure to misinformation in fitness environments.

An interesting observation of this study concerns the fact that 11.7% of users are unaware of whether their supplement actually contained *Tt* or protodioscin. In this concern, although the study did not directly assess participants’ reading ability, label comprehension skills, or general health literacy, we interpreted this occurrence as respondents’ self-reported uncertainty regarding the composition of *Tt*-containing products, which we considered indicative of limited awareness of product content rather than a direct measure of label comprehension or knowledge. In our opinion, *Tt* consumption may not always be a fully informed practice, but rather the consequence of marketing-driven expectations and symbolic beliefs in “natural” muscle enhancement. These observations align with recent research showing that supplement users frequently overestimate efficacy, underestimate risks, and rely on commercial claims instead of evidence-based information [34,35,36]. Our results extend this perspective by demonstrating how ignorance of ingredient composition exposes consumers to pharmacologically active substances without awareness or understanding, particularly among individuals purchasing products via unregulated online sources.

Overall, *Tt* and *Tt*-containing products are generally perceived as ergogenic supplements, in line with the widespread belief that they are capable of enhancing testosterone secretion and improving physical performance. This understanding is discordant with current scientific research that does not demonstrate any appreciable anabolic or ergogenic effect in people [1,27,29,37]. Indeed, while early preclinical animal studies suggested androgenic-like effects of *Tt*, largely as a result of saponin Protodioscin effect [15,16,17], subsequent controlled human clinical trials all reported infinitesimal or non-statistically significant findings. For instance, Fernández-Lázaro et al. [37] reported no increase in hormonal response, body composition, or performance after six weeks of *Tt* supplementation in trained CrossFit athletes, while a systematic review by the same authors confirmed the absence of apparent advantages in sport and health biomarkers [1]. These findings underscore a persistent discrepancy between popular perception and evidence-driven knowledge, uncovering the processes of placebo effects, expectancy bias, and advertising messages that organize consumer decision making.

The notion that “natural” can be equated with “safe” or “effective” appears deep-seated among supplement consumers, particularly within gym settings where social influence and casual expertise are strong [27]. These assumptions can lead to unsupervised consumption and risk minimization, like product adulteration, dosing variations, and potential interactions with other supplements or medications [15]. Notably, a high percentage of this study’s participants (11.7%) did not know whether their supplements contained *Tt*. This occurrence suggests a passive consumption pattern where users may rely on marketing or peer recommendation rather than informed decision-making. In the context of our study’s objectives to evaluate potential misuse, this behavior increases the susceptibility to adverse health effects due to unintentional drug-supplement interactions or the consumption of contaminated products (‘inadvertent doping’). Therefore, low label awareness is not merely an informational gap, but a behavioral predictor of unsafe supplementation practices. This issue is further compounded by the high heterogeneity in the composition of commercial *Tt* products, which changes according to factors such as plant source, extraction methods, and standardization processes [4,5,8,15]. Moreover, quality control issues, including adulteration with unlisted pharmacologically active ingredients or synthetic analogs, have been reported [1], suggesting the need for stricter regulatory surveillance and more transparent labeling.

Motivational analysis shows that the primary reason for *Tt*-supplement intake was enhancement of performance. This supports existing literature where performance optimization has been noted as a dominant factor for supplement use by recreational athletes [38]. Many users seem to perceive *Tt* as a natural, legal substitute for anabolic steroids, with the belief that it is safe, even without adequate evidence of effectiveness [27]. These attitudes are generally supported by social media, peer advising, and fitness culture, that valorize supplementation as a legitimate path to improvement.

Consequently, the consumption of supplements is normalized as part of the training schedule in both sexes and across all age ranges, as suggest also by other European research [29,33], raising ethical and public health concerns over medicalization of sport and performance.

While the prevalence of anabolic-androgenic steroid (AAS) use in our data was low, the data revealed a notable preference for online purchasing, especially among individuals motivated by esthetic goals. This behavior poses serious risks, as online markets for performance-enhancing substances often lack adequate regulation and quality assurance [2,33]. The anonymity and accessibility of these channels may lower barriers to experimentation and facilitate access to counterfeit or contaminated products. This phenomenon fits within the broader paradigm of image- and performance-enhancing drug use, where the pursuit of rapid physical transformation often overrides considerations of safety and health. Addressing such behaviors requires targeted interventions focused on digital literacy, consumer awareness, and the regulation of e-commerce platforms involved in supplement distribution.

From a public health perspective, the findings of this study suggest the potential value of coordinated approaches that integrate education, regulation, and risk communication. Improvements in labeling practices, clearer disclosure of active compounds, and product authenticity certification may contribute to enhanced consumer protection. In parallel, educational initiatives aimed at gym users and athletes could promote evidence-based awareness, including consideration of the limited effectiveness and possible risks associated with insufficiently tested herbal supplements. As *Tt* is absent in the 2025 World Anti-Doping Agency (WADA) Prohibited List [28,39], it is likely to be viewed by athletes as a harmless performance-enhancer. However, such reasoning blurs the distinction between legal enhancement and doping intent and suggests the need for preventive measures and education campaigns aimed at so-called “grey-zone” drugs—that is, drugs that are not banned but are widely abused due to insidious manipulation.

Despite the interesting observations raised from this study, on a circumscribed but unstudied population, some limitations should be noted.

First, the study employed a non-probabilistic convenience sampling strategy limited to fitness centers with prior collaborative agreements, resulting in an overrepresentation of urban facilities, predominantly from Northern and Central Italy. Consequently, the observed prevalence of *Tt* consumption may not fully reflect the broader Italian gym population. Second, data were self-reported and susceptible to recall inaccuracies and social desirability bias, particularly regarding sensitive behaviors associated with performance enhancement. Third, the cross-sectional design precludes causal inference; motivation and *Tt* consumption may reinforce each other over time, and temporality cannot be established. In addition, the low proportion of events per variable represents a statistical limitation which emphasize the magnitude of the odds ratio, which is approximately 18.5 times. Fourth, although *Tt* doses were standardized based on declared product labels, their actual chemical content could not be analytically verified, limiting the ability to assess biological exposure.

Despite these limitations, the study provides valuable epidemiological insight into *Tt* use in real-world fitness settings, capturing motivational and behavioral risk patterns not otherwise evident in clinical research.

Suggestion for future research should therefore use longitudinal and mixed-method designs to assess temporal change in supplement consumption and analyze psychological, sociocultural, and online drivers of use. The combination of biomarker-based evaluation and label confirmation may enhance the accuracy of data and help deliver a more accurate assessment of exposure, efficacy, and safety. Further studies must investigate dose–response relations, interactions between drugs and pharmacological effects, and the interaction between knowledge, motivation, and risk perception, thus informing the development of personalized preventive and educational policies.

## 5. Conclusions

*Tt* use in Italian gym environments is a low-prevalence yet highly performance-driven behavior, reflecting the influence of commercial claims, symbolic beliefs in “natural anabolic boosters,” and limited label literacy. Consumers motivated by athletic performance were over 18 times more likely to currently use *Tt* than users seeking other benefits, highlighting a motivational factor associated with escalating reliance on ergogenic aids. These findings reinforce the need for evidence-based prevention in sports nutrition, emphasizing clear education on supplement composition, efficacy, and regulatory status. Gyms and fitness organizations should adopt communication strategies that address misconceptions about “natural enhancers,” promote critical appraisal of commercial claims, and support informed consumer decision-making. Moreover, regulatory surveillance of online supplement markets remains essential to safeguard consumers against poorly labeled or misleading products. Through educational and market-oriented interventions, sports nutrition policies can better align public perception with scientific evidence and mitigate the behavioral risks associated with performance-oriented supplementation.

## Figures and Tables

**Table 1:** Characteristics of the Total Sample (N = 510).

Variable	Category	n	%
Gender	Male	240	47.1
	Female	250	49.0
	Prefer not to answer	20	3.9
Age	18–24 years	151	29.6
	25–34 years	174	34.1
	35–44 years	88	17.3
	≥45 years	97	19.0
Geographical Area	North-West	35	6.9
	North-East	25	4.9
	Central	355	69.6
	South and Islands	87	17.1
	Extra ITA	3	0.6
	Not reported	5	1.0
Sport activity	Yes, competitive level	59	11.6
	Yes, train regularly at the gym	336	65.9
	No	57	11.2
	Other	39	7.6
	No response	19	2.7
Weight (kg)	Mean (SD)	69.74 (13.85)	
Height (cm)	Mean (SD)	171.46 (8.92)	

**Table 2:** Characteristics of Supplement Users (N = 189).

Variable	Category	n	%
Gender	Male	92	48.7
	Female	89	47.1
	Prefer not answer	8	4.2
Age	18–24 years	39	20.6
	25–34 years	68	36.0
	35–44 years	39	20.6
	≥45 years	43	22.8
Geographical Area	North-West	11	5.8
	North-East	10	5.3
	Central	136	72.0
	South and Islands	31	16.4
	Not reported	1	0.5
Sport activity	Yes, competitive level	25	13.2
	Yes, train regularly at the gym	137	72.5
	No	10	5.3
	Other	16	8.5
	No response	1	0.5
Reason for using supplements	To improve performance	67	35.4
	To compensate for nutritional deficiencies	52	27.5
	To improve physical appearance	36	19.0
	Other	34	18.0
Weight (kg)	Mean (SD)	71.03 (13.44)	
Height (cm)	Mean (SD)	171.7 (8.96)	

**Table 3:** Multivariable Logistic Regression Predicting *Tt* Use.

Associated Factors	Adjusted or (95% CI)	*p*-Value
Motivation for supplement use (non performance vs. performance)	0.054 (0.006–0.461)	0.008
Sex (male vs. female)	0.859 (0.354–2.084)	0.918
Age	0.979 (0.934–1.026)	0.370

## Data Availability

The data presented in this study are available on request from the corresponding author due to privacy/ethical reasons in compliance with the General Data Protection Regulation (GDPR).

## Supplementary Materials

The following supporting information can be downloaded at: https://www.mdpi.com/article/10.3390/nu18020253/s1, Material S1: Survey Instrument.
