# Supplementary material for: Awareness and Risk Behaviors Associated with Tribulus terrestris (Tt), Dietary Supplements, and Anabolic Steroids: Evidence from an Italian Questionnaire-Based Study
Source: Nutrients. 2026 Jan 13;18(2):253. doi: 10.3390/nu18020253 (PMC12844756; doi:10.3390/nu18020253)
Supplement: Supplementary file 1 [file nutrients-18-00253-s001.zip › nutrients-4080988-supplementary.pdf]

Supplementary Materials S1: Survey Instrument

Awareness and Risk Behaviors Associated with *Tribulus terrestris* (Tt), Dietary Supplements, and Anabolic Steroids: Evidence from an Italian Questionnaire-Based Study

Note: This questionnaire was derived and adapted from validated survey forms previously employed by the Italian National Institute of Health (ISS) for national doping and supplementation surveillance campaigns. The survey was anonymous and participation was voluntary.

Section 1: Socio-Demographic and Anthropometric Information

1. Age (years): [_______]

2. Gender: [ ] Male [ ] Female [ ] Prefer not to answer

3. Place of Residence (City/Region): [____________________]

4. Height (cm): [_______]

5. Weight (kg): [_______]

Section 2: Sports Activity

6. Do you practice sports activity? [ ] Yes, at a competitive level [ ] Yes, I train regularly at the gym (recreational/amateur) [ ] No [ ] Other: ____________________

Section 3: Dietary Supplement Use

7. Usually, where do you purchase your dietary supplements? [ ] Internet [ ] Pharmacy [ ] Sports nutrition store [ ] Gym [ ] Other: ____________________ [ ] I do not use supplements

8. What is the primary reason for using supplements? [ ] To improve physical performance [ ] To compensate for nutritional deficiencies [ ] To improve physical appearance (aesthetics) [ ] Other: ____________________

9. Please indicate the quantity of supplements consumed in the last week (if applicable):

- Number of capsules: [_______]
- Number of sachets: [_______]
- Number of drops: [_______]

Section 4: *Tribulus Terrestris* and Specific Substances

10. In the last 6 months, have you used supplements containing *Tribulus terrestris*? [ ] Yes [ ] No [ ] I don't know/I am not sure if my supplements contain it

11. In the last week, have you used supplements containing *Tribulus terrestris*? [ ] Yes [ ] No

12. In the last 6 months, have you used anabolic-androgenic steroids (AAS)? [ ] Yes [ ] No

13. If yes, where did you purchase them? [ ] Internet [ ] Gym environment/Peers [ ] Other
